# Lived experience of people with adrenocortical carcinoma and associated adrenal insufficiency

**DOI:** 10.1002/edm2.341

**Published:** 2022-06-06

**Authors:** Phillip Yeoh, Wladyslawa Czuber‐Dochan, Simon Aylwin, Jackie Sturt

**Affiliations:** ^1^ Florence Nightingale Faculty of Nursing Midwifery & Palliative Care. King's College London London UK; ^2^ Department of Endocrinology & Diabetes The London Clinic London UK; ^3^ Department of Endocrinology King's College Hospital London UK

**Keywords:** adrenal insufficiency, Adrenocortical carcinoma, lived experience, systematic review, wellbeing

## Abstract

**Introduction:**

Adrenocortical carcinoma (ACC) is a rare cancer with an annual incidence of 0.7–2 cases per million population and 5‐year survival of 31.2%. Adrenal insufficiency (AI) is a common and life shortening complication of ACC, and little is understood about how it impacts on patients' experience.

**Objective:**

To understand patients' lived experience of the condition, its treatment, care process, impact of AI on ACC wellbeing, self‐care needs and support.

**Methods:**

Systematic review of MEDLINE, EMBASES, CINAHL, PsycINFO and Open Grey for studies published until February 2021. All research designs were included. The findings underwent a thematic analysis and narrative synthesis. Studies quality was assessed using mixed method assessment tools.

**Results:**

A total of 2837 citations were identified; 15 titles with cohort, cross‐sectional, case series and case report study designs met the inclusion criteria involving 479 participants with adrenal insufficiency secondary to adrenocortical carcinoma (AI/ACC). Quantitative research identified impacts of disease and treatment on survivorship, the burden of living with AI/ACC, toxicity of therapies, supporting self‐care and AI management. These impact factors included adjuvant therapies involved and their toxicities, caregivers/family supports, healthcare and structure support in place, specialist skill and knowledge provided by healthcare professional on ACC management. No qualitative patient experiences evidence was identified.

**Conclusion:**

ACC appears to have high impact on patients' wellbeing including the challenges with self‐care and managing AI. Evidence is needed to understand patient experience from a qualitative perspective.

## Graphical abstract

ACC appears to have high impact on lived experience of patients including their wellbeing and AI management. Further qualitative study is needed to understand the interactions of these two conditions, it's influences on patients' journey and survivorship.

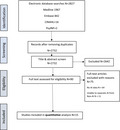

## 1 INTRODUCTION

### 1.1 Background

Adrenocortical carcinoma (ACC) is a rare and usually aggressive cancer with poor outcomes for many patients. Its incidence worldwide is estimated to be around 0.7–2 cases per million per year in adult populations.^1^, ^2^ The median age at diagnosis is 56 years with women and white Caucasians being more frequently affected.^1^, ^3^ Patients with ACC often present with secretory tumours leading to Cushing's syndrome and/or androgen excess.^4^, ^5^ Survival depends on staging at presentation; overall median survival rate can be as low as 17 months.^1^ The 5‐year survival rate is 31.2%, and this has failed to improve from data collected between 1983–2009^1^, ^2^, ^6^ despite advances in treatment.

Existing evidence indicates that people with adrenal disease have reduced quality of life irrespective of hypo, or hyperfunction.^7^ People with ACC also have more unhealthy days, worse quality of life and mood scores, and higher odds of depressive symptoms compared to those with benign non‐functioning tumours or benign hormonal disorders such as primary hyperaldosteronism, Cushing's syndrome, congenital adrenal hyperplasia and Addison's disease.^8^ Clinical guidelines on the management of ACC acknowledged that there was a lack of evidence understanding the impact of ACC on an individual's life.^9^

Surgical resection followed by adjuvant therapy with the adrenolytic agent mitotane has been the mainstay of treatment since the 1970s.^9^, ^10^ There is a role for chemotherapy and radiotherapy in addition. Patients with ACC treated with mitotane commonly develop toxicity, including adrenal insufficiency (AI) and individuals with pre‐existing cortisol excess may become glucocorticoid insufficient post‐operatively. Several studies have found that 90/407 (22%) patients with ACC developed AI peri‐ or post‐operatively following surgery and this incidence increased to 50% in the subgroup with cortisol secreting ACC.^10^, ^11^ Mitotane represent the first‐line post‐operative treatment with its adrenolytic properties.^12^ Patients on long‐term mitotane adjuvant therapy start to show biochemical evidence of AI within weeks to months into mitotane therapy.^13^ Mitotane also induces the drug‐metabolizing enzyme CYP3A4, increasing drug metabolism including that of glucocorticoids used to treat AI.^13^ Several studies reported that higher replacement dose of glucocorticoid was required to treat AI related to ACC (AI/ACC) and to prevent adrenal crisis, compared with other causes of AI.^14^, ^15^, ^16^ Whilst qualitative data on living with ACC is available, a scoping review revealed no qualitative patient experience evidence for living with AI related to ACC.^17^ This systematic review aimed to understand patient lived experience and wellbeing in AI secondary to ACC using evidence from a variety of study designs and thereby extract evidence related to the impact and management of this condition on patient wellbeing to inform a holistic approach to person‐centred care.

### 1.2 Objectives

To systematically review the evidence to answer the following questions:

1. What is the patients' experience of living with ACC in relation to AI?
2. How is patients' wellbeing and lived experience impacted by the condition, its treatment and management regimen?

## 2 METHODS

### 2.1 Search strategy

MEDLINE, EMBASES, CINAHL, PsycINFO and Open Grey electronic databases were searched for completed and ongoing studies of any design from database inception until February 2021. The study was registered in PROSPERO (IDCRD42020175255 registered on 28/4/2020) using key terms, Medical Subject headings, sub‐headings and search variables (see Table S1).

### 2.2 Inclusion and exclusion criteria

Inclusion criteria were empirical studies of any design, published in English, involving patients of any age, including children, with AI/ACC. Paediatric studies were included to understand the perspectives of carers/parents. Studies focused on patients with ACC management including lived experience and wellbeing outcomes were included where data on a subset of people with secondary AI were reported separately. Exclusion criteria were non‐human studies.

### 2.3 Screening

**FIGURE 1:**
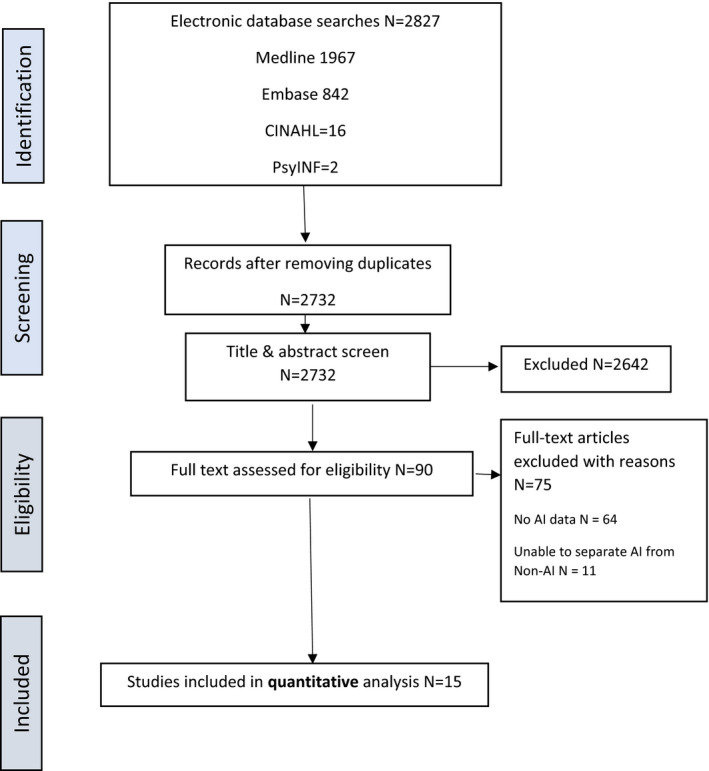
PRISMA flow chart

Identified citations were exported to Endnote. A total of 2827 citations were identified; 95 duplicates were removed; 2732 titles and abstracts were screened against eligibility criteria. No grey literature was identified for inclusion. 2642 titles were excluded at the title and abstract screen, 90 eligible full text papers were identified by investigator PY. 15 papers met the inclusion criteria (see PRISMA diagram Figure 1).

### 2.4 Data extraction and synthesis

Results from the included papers were extracted to a table and include treatment regime, survivorship, side effects and toxicity experienced, patient reported outcomes, impact of clinical management and supports provided. Due to the heterogeneity of study designs, different population and assessment tools, meta‐analysis was not possible and instead, narrative convergent synthesis was utilized to analyse the evidence.^18^

### 2.5 Quality assessment

The 15 studies (quantitative and mixed methods) were appraised by solo researcher (PY) using mixed methods appraisal tool^18^, ^19^, ^20^ (see Table S2). This quality appraisal tool found 9/15 (60%) studies met all the criteria in the assessment tool whereas 6/15 (40%) met 80%.

## 3 RESULTS

### 3.1 Study characteristics

The 15 studies included a total of 479 patients who had AI/ACC: 196 males and 283 females. The median age of the adult patients were 45 years with the youngest age 2 years and the oldest one age 79.6 years. 87% of the included patients were white Caucasian.^22^

Studies were undertaken in Italy (*n* = 5),^13^, ^24^, ^25^, ^30^, ^31^ France (*n* = 1),^28^ Netherlands (*n* = 1),^23^ Pan European Union involving Italy, France, Germany, and Netherlands (*n* = 1),^29^ USA (*n* = 4),^21^, ^22^, ^26^, ^27^ Australia (*n* = 1),^16^ Brazil (*n* = 1)^14^ and Canada (*n* = 1).^15^ The study designs consisted of nine cohort studies,^13^, ^14^, ^21^, ^22^, ^24^, ^25^, ^26^, ^28^, ^29^ four case reports,^15^, ^16^, ^30^, ^31^ one cross‐sectional^23^ and one case series.^27^ All studies reported quantitative data published between 1994 and 2020. Cross‐sectional or cohort studies had sample sizes ranging from 11–122, whereas case series had six patients and case reports had one to two patients (Table 1, Table S3).

**TABLE 1:** Characteristics of the included studies (in chronological order)

Author (year), reference no, study design	Aim of study	Study population	Main results/theme
Haak et al (1994)^23^ Cross sectional	To evaluate the relevance of therapeutic level of mitotane on survival	96 adults	Patients presented with hormonal excess, pain, and changes in physical appearance. Only 48% managed to achieve target level due to toxicity effects. Patients with inoperable ACC died within 18 months
Williamson et al (2000)^22^ Cohort	To evaluate the response rate and toxic effects to mitotane	45 adults	11% of cohort were unable to work. 4% died of infection and respiratory distress. Up to 82% of patients experienced mild to disabling grade of toxicity effects
Abraham et al (2002)^26^ Cohort	To determine the efficacy of chemotherapy with oral mitotane therapy	36 adults	All cohorts were restricted in their physical activities. They had physical symptoms, various metastasis, and adjuvant therapy. They required over five cycles of chemotherapy to achieve a median survival of 34.3 months. 66% experienced chemotherapy and 47% had mitotane toxicities
Berruti et al (2005)^24^ Cohort	To investigate the activity of chemotherapy plus mitotane	72 adults	39% of cohorts were restricted in physical activity, 15% unable work and 3% were limited in self‐care. 68% had hormone hypersecretion and only 52.5% managed 6 cycles of chemotherapy. 90% experience toxicity of mitotane resulted in 6.9% stopping mitotane
Zancanella et al (2006)^14^ Cohort study	To define a mitotane dose that maintains therapeutic plasma levels	11 children	All patients experienced mitotane toxicity One patient died from adrenal crisis and 45% experienced adrenal crisis during the study. Carers provide direct care and support to patients during ACC treatments
Daffara et al (2008)^13^ Cohort study	To assess the unwanted effects of mitotane	17 adults	37% experienced tumour recurrence, 17% died of ACC progression and it took 9 months for them to achieve therapeutic mitotane level. Up to 71% experienced mitotane toxicity. They also developed AI, hypothyroidism, hypercholesterolaemia and men had low testosterone and required treatments
Sperone et al (2010)^25^ Cohort study	To access the activity and toxicity of chemotherapy	28 adults	36% of cohorts were restricted in physical activity, 11% unable to work. They required adjuvant therapy and additional treatments. 50% had hormonal excess. Treatments lead to severe or disabling grade of toxicity effect. Overall survival was 9.8 months
Lacroix (2010)^15^ Case report	To describe the challenges of ACC clinical management	1 adult	Presented with signs and symptoms of ACC. Interventions involved surgery, chemotherapy, adjuvant mitotane leading to AI and toxicity effects Required MDT and palliative care support at the end
Meuclère‐Denost et al (2012)^28^ Cohort study	To evaluate the effects of mitotane	22 adults	36% had restricted physical activity, they experienced hormone excess, surgery such as nephrectomy and palliative treatment with mitotane with only 45% were able to achieve therapeutic mitotane level. 50% discontinued mitotane due to toxicity and up to 91% experienced mild to disabling grade of toxicity. 18% permanently discontinued due to tumour progression. They also received normal salt diet and AI education
Terzolo et al (2013)^29^ Cohort study	To compare recurrence free survival in patient who achieved therapeutic mitotane level	122 adults	Despite disconcerting efforts, only 53% were able to achieve target mitotane concentration. 24.5% discontinued mitotane treatment by choice and toxicity effects. 47.5% experienced recurrence and 27% die from ACC
Lerario et al (2014)^21^ Cohort study	To assess the efficacy of the combination of the IGF1R inhibitor cixutumumab.	20 adults	40% were unable to perform physical strenuous activity. One (5%) death with multiorgan failure, and 2 (10%) severe cases of hyperglycaemia
Fancellu et al (2014)^30^ Case report	To describe patient experience	1 adult	Sibling had genetic condition predispose him to develop ACC. He then developed hormonal excess required surgery and mitotane adjuvant therapy for his ACC
Kanjanapan et al (2015)^16^ Case report	To illustrate clinical management of ACC	1 adult	Presented with pain, virilisation, and androgen excess. She had surgery and mitotane followed by glucocorticoid replacement therapy. Following an adrenal crisis precipitated by missed glucocorticoid dose, steroid education on sick day management was provided. After 2 years of mitotane therapy, she elected to stop mitotane due to its toxicity and remained on glucocorticoid replacement. 7 months later, she had another adrenal crisis precipitated by viral illness resulted in cardiac arrest and death
Head et al (2019)^27^ Case series	To assess efficacy of immunotherapy	6 adults	83% were unable to perform physical strenuous activity. They had hormonal excess and multiple surgeries. They also experienced mild to severe adverse effects of immunotherapy. All patients had hypothyroidism
Muratori et al (2020)^31^ Case study	To describe ACC and its AI management	1 adult	Presented with pain, hypertension, diabetes mellitus, obesity and left retroperitoneal mass of 18 × 12 cm. Had surgery, radiotherapy, adjuvant mitotane and glucocorticoid replacement therapy. Unable to sustain therapeutic mitotane level and decided to discontinue. His AI eventually. Few months later, AI recurred following an anaphylaxis event and this was 3 years after stopping mitotane

Treatment pathways were intensive; 448/479 (93.5%) of the patients included in all 15 studies had previously undergone surgery. Three studies recorded a frequency of surgeries, and out of 106 participants, 31 had two or more surgeries.^24^, ^25^, ^27^ The multiple surgical experiences were illustrated in case studies.^15^, ^16^ In four studies that reported tumour metastasis, 73 out of 126 patients had more than one site.^21^, ^24^, ^25^, ^27^ Similar pattern was noted in nine studies that recorded tumour staging, 283 out of 297 patients had staging II or above.^13^, ^14^, ^22^, ^24^, ^27^, ^28^, ^29^, ^30^, ^31^

### 3.2 Study themes

Five themes were developed from data synthesis with 15/15 (100%) studies in survivorship, 14/15 (93%) in burden of living of ACC, 13/14 (87%) in toxicity of therapies, 12/15 (80%) supporting self‐care and 7/15 (47%) in AI management (see Table 2).

**TABLE 2:** Themes created from 15 studies

Studies	Narrative analysis themes				
	Survivorship	Burden of living with ACC	Toxicity of therapies	Self‐care needs & support	AI management
Haak et al (1994)	√	√	√		
Williamson et al (1999)	√		√	√	
Abraham et al (2002)	√	√	√	√	
Berruti et al (2005)	√	√	√	√	
Zancanella et al (2006)	√	√	√	√	√
Daffara et al (2008)	√	√	√	√	√
Sperone et al (2010)	√	√	√	√	
Lacroix (2010)	√	√	√	√	√
Meuclère‐Denost et al (2012)	√	√	√	√	√
Terzolo et al (2013)	√	√	√		
Lerario et al (2014)	√	√	√	√	
Fancellu et al (2014)	√	√			√
Kanjanapan et al (2015)	√	√	√	√	√
Head et al (2019)	√	√	√	√	
Muratori et al (2020)	√	√		√	√
Total	15	14	13	12	7

#### 3.2.1 Survivorship

The treatment pathways for ACC were presented as challenging and various interventions have significant impact on patients' clinical outcomes. These findings were supported by several case studies which illustrated the complex patients' journeys.^15^, ^16^, ^31^ The early part of ACC pathways was dominated by diagnosis and therapeutic efforts, particularly with surgery as the first choice of treatment. Three studies that looked at outcomes in patients with inoperable disease or not responding to chemotherapy and mitotane, median survival in all patients did not exceed 18 months.^23^, ^25^, ^26^ Surgical resection with suboptimal mitotane levels achieved median survival of 98 months.^29^ Adding to these challenges, several studies reported tumour recurrence between 75/150 (50%) of patients.^13^, ^14^, ^29^ Only 66/192 (34.4%) of patients who received chemotherapy achieved positive responses.^14^, ^22^, ^24^, ^25^, ^26^ At the end of the studies, 88/267 (33%) of patients died.^13^, ^14^, ^22^, ^24^, ^29^ Mitotane with chemotherapy achieved an overall survival of 48.6% at 2‐years, then dropped to 14% at 5‐years.^24^ One case study suggested that patient survival beyond 2 years was influenced by the consequences of coping with this condition.^16^ Patients with ACC need to learn to cope with the disease and adhere to treatment progression while preparing for the possibility of facing palliative care.^14^, ^15^, ^16^, ^25^, ^26^, ^28^

#### 3.2.2 The burden of living with AI/ACC

The burden patients faced following their diagnosis were not necessarily related to AI. Over 50% of their symptoms were related to ACC with hormonal excess leading to changes in their physical appearances.^15^, ^16^, ^23^, ^24^, ^25^, ^30^, ^31^ Patients' perception of these changes was not reported. In the absence of qualitative evidence, four case studies offer limited data regarding the patient's journey with numerous hospital visits and admissions starting from clinical presentations to diagnostic, therapeutic, surveillance and late effects phases.^15^, ^16^, ^30^, ^31^

Baseline performance status of 215/229 patients were recorded.^21^, ^22^, ^24^, ^25^, ^26^, ^27^, ^28^ Performance status quantifies cancer patients' level of functioning, general wellbeing, and their ability to care for themselves with score 0 as fully active, score 4 as completely disabled and score 5 as death.^32^ Over the years, performance status score has been adopted by World Health Organization and The Eastern Cooperative Oncology Group (ECOG).^33^, ^34^ Over 52% of the patients reported performance status scoring from 1–3, indicating physical activity restriction to limited self‐care.^21^, ^22^, ^24^, ^25^, ^26^, ^27^, ^28^ These impairments demonstrated the negative impact of ACC on wellbeing of patients living with this condition.

Mitotane initiation to achieve therapeutic levels was challenging for some patients. It was reported that mitotane absorption was unpredictable.^14^, ^15^, ^16^, ^31^ One study was unable to find any significant correlation between the cumulative mitotane dose and the plasma mitotane level.^28^ It took up to 15 months for some patients to achieve therapeutic mitotane levels, 18% patients discontinued mitotane treatment and 50% required transient discontinuation.^13^, ^14^, ^22^, ^23^, ^24^, ^28^ The unpredicted side effects of mitotane coupled with its discontinuation created additional mental challenges of living with AI/ACC.

Carers of ACC patients were faced with extensive responsibilities such as providing personal care, medication adherence, symptoms management, understanding toxic drug side effects, learning to identify the need for glucocorticoid adjustment, knowing when to intervene to prevent adrenal crisis or administering hydrocortisone injections if adrenal crisis occurred.^14^

#### 3.2.3 Toxicity of therapies

Patients who decided to undergo ACC treatments were faced with the symptoms of treatment toxicities which could have profound impact on their lives.^13^, ^14^, ^15^, ^21^, ^22^, ^23^, ^24^, ^25^, ^26^, ^27^, ^28^, ^29^ Those who endured chemotherapy for ACC were given a median of five to six cycles (range 1–16 cycles), respectively^24^, ^26^ with only 52.8% of the patients completing the treatment plan of six cycles.^24^ Earlier findings reported that patient survival was influenced by achieving mitotane therapeutic levels (*p* = 0.01) and total resection at first surgery (*p* < .001).^23^ A later study found that after adjustment for sex, age at diagnosis, ENSAT (European Network for the Study of Adrenal Tumors) stage, hormone secretion, Weiss score and mitotic index, the patients who maintained target mitotane concentrations of 14 mg/L or higher showed a significantly reduced risk of recurrence (adjusted HR, 0.418; 95% CI, 0.22–0.79; *p* = .007) while the risk of death was not significantly altered (adjusted HR, 0.59; 95% CI, 0.26–1.34; *p* = .20).^30^ Some researchers reported that adverse effects were to be expected, transient, tolerable and could be resolved by dose adjustment or temporary discontinuation of treatment.^15^, ^16^, ^28^, ^29^ However, patients' decisions to terminate treatment were reported to be driven by side effects, tumour progression and treatment choice, while their emotional wellbeing was not explored in depth.^16^, ^23^, ^24^, ^28^, ^29^, ^31^ The fear of living with AI/ACC was not explored in these studies but in the case studies, the mental challenges were apparent in facing unpleasant outcomes and death.^15^, ^16^, ^30^ These experiences demonstrate that fear, anxiety, pain and dealing with own's sense of mortality are common features of acute phase of cancer survival which could have been explored concomitantly.^35^

#### 3.2.4 Supporting self‐care

Self‐care needs and deficits in patients living with AI/ACC was addressed in two of the case studies.^14^, ^16^ A patient was ‘given education’ on glucocorticoid replacement sick day rules following her first adrenal crisis.^16^ The format and delivery of this education was unclear. Other case reports failed to describe if AI education to improve self‐care was provided.^30^, ^31^

Complex self‐care needs in relation to their oncological treatment and self‐care activities were evidenced in five studies.^13^, ^14^, ^23^, ^28^, ^29^ For example, mitotane was recommended to be taken orally with meals containing fat to improve absorption.^14^, ^23^, ^28^ Some studies utilized serum mitotane levels to guide mitotane dose titration, allowing patients to return to a lower dose or discontinued temporarily when patients experienced unacceptable side effects.^13^, ^28^, ^29^ A patient‐centred approach managed by physicians involving monthly assessment was guided by clinical tolerance, biochemical test results and mitotane levels.^28^ Patient visits were supported by continuity of care with the same physicians to detect subjective symptoms that might have occurred during treatment.^13^ Continuous support was offered to patients and their primary care physicians by means of phone and email contacts to cope with side effects. To mitigate side effects and improve self‐care, antiemetics were prescribed routinely as the standard care.^28^ Patient care was supported by regular imaging, physical examination, laboratory evaluation, monitoring of mitotane concentrations and hormonal assessment.^29^ In one case study, initiation of mitotane therapy after ACC diagnosis was preceded by multidisciplinary and psychological support followed by palliative care.^15^ These established pathways provided a structure allowing patients and their families to engage with their clinical care, while adopting a patient‐centred approach.

#### 3.2.5 AI management

Patients started to develop AI within 3 months of taking mitotane^13^ and in one study, all six patients became adrenal insufficient within 16 weeks.^27^ The impact of AI could last several years after stopping mitotane.^31^ Symptoms of adrenal crisis were experienced by 5/11 (45%) of patients who required prompt treatment with intravenous hydrocortisone and hydration.^14^ Hydrocortisone omission during intercurrent illness resulted in 2/12 (16.7%) of patients' deaths from adrenal crisis.^14^, ^16^

Patient and families were unaware of the need to adjust glucocorticoid/steroid dosage required during illness.^14^, ^16^ This was highlighted by patients' experience where AI managements were complicated by the effects of mitotane and treatment modalities.^15^, ^31^ To reduce the risk of developing adrenal crisis, people with AI related to ACC need to be educated on the importance of glucocorticoid replacement.^13^, ^15^, ^16^ Unfortunately, details of the education programs to address AI were absent among the reviewed studies.^13^, ^14^, ^15^, ^16^, ^28^, ^30^, ^31^ Adding to this, many of the symptoms related to adrenal crisis resembled mitotane, chemotherapy or immunotherapy toxicities making patient AI focus approach most challenging.^13^, ^14^, ^15^, ^16^, ^21^, ^22^, ^23^, ^24^, ^25^, ^26^, ^27^, ^28^, ^29^

## 4 DISCUSSION

### 4.1 Main findings

This systematic review demonstrates the limited evidence that underpins the effectiveness of support for self‐care or management of survivorship in the light of excessive treatment burdens. The treatment, care process, quality of life, wellbeing, self‐care needs and support did not provide rich narratives to reflect patients' overall experiences and wellbeing. This review identified the significant toxicity that people with AI/ACC endure, high healthcare burden and consequences related to AI/ACC followed by poor survival, self‐care shortfall, impact on wellbeing and education needs. Treatment and care process were dominated by diagnosis and therapeutic efforts. Fear and concerns with treatment and disease progression were not well addressed and consequently, give rise to research questions for the next empirical stage.

### 4.2 Findings compared with wider evidence

A systematic review on quality of life of 323 patients with ACC concluded that they have worse quality of life scores compared with the general population.^36^ Other studies found standard ACC treatments failed to improve the quality of life of patients, with 178/340 (52%) patients having ECOG scores from 1 to 4.^37^, ^38^ Patients with functional ACC were found to have other preoperative comorbidities such as diabetes, hypertension, congestive heart failure, obesity and coagulopathy, leading to post‐operative complications, increased Charlson comorbidity index, tendency toward emergency operations and longer hospitalizations.^3^ The impact of chronic comorbidities in ACC requires further investigations to understand the relevant contribution to overall morbidity among these coexisting conditions.

Recent qualitative interviews with 10 ACC patients identified four domains related to their wellbeing.^17^ They were physical complaints, mental consequences, social consequences and functional limitations. These concerns related to feeling insecure between radiological procedures, limitations with daily activities and mobility, experiences with healthcare system and professionals. AI data were not analysed separately from non‐AI hence the study was not included in this systematic review. However, the study also found patient experiences and partner perspectives influences wellbeing. The effect of a supportive partner was identified by another study which found marital status had positive association (*p* = .008) with ACC survival.^39^ It has been suggested that this might be attributed by increased peer support for access to healthcare, treatment adherence and willingness to seek help. These findings align with the themes in our systematic review and reinforce the case for qualitative research in people with AI/ACC.

Healthcare professionals recognized that preventing adrenal crisis is one of the main goals for patient on AI treatment.^40^ AI education is paramount for patients and their caregivers to anticipate, recognize and intervene early to prevent or reduce the occurrence of adrenal crisis.^40^, ^41^ Patients with AI related to ACC expressed their frustration that simple flu could lead to hospitalization.^17^ Patients with non‐ACC related AI who were given AI education and were able to self‐inject glucocorticoid during emergency had quicker symptoms improvement and were more likely to get treated in an outpatient setting compared with those who did not self‐inject.^42^ Similarly, those who experienced adrenal crisis were more likely to adjust their glucocorticoid dose when required compared to those without.^43^, ^44^ Patients with AI without ACC who received AI education self‐reported that they were better in recognizing signs and symptoms of their adrenal crisis immediately after the training rather than 6–9 months afterwards.^44^ Thus, there is a need to provide a structured education, continuous training and support to AI related to ACC by drawing from the existing knowledge, skills and modes identified to support self‐efficacy and self‐care for people on AI treatment.^41^, ^45^

People on AI treatment with specific beliefs about the necessity of AI replacement including its concerns about adverse effects were found to have more negative illness perceptions.^46^ Endocrine nurses can play a pivotal role in addressing these beliefs and concerns through AI/ACC education, supporting their physical symptoms, addressing emotional, mental, and psychological needs and wellbeing by coaching and signposting.

Increasingly, people with cancers are advocating for improved information resources for cancer care.^47^, ^48^, ^49^ A feasibility study explored the delivery of a Survivorship Care Planning Program by using an information booklet, telephone coaching and mobile phone app‐based integrated activity tracker, for breast cancer survivors who reported improvement in fatigue, health distress, self‐care knowledge and emotional wellbeing.^50^ However, the study did not find improvement on breast cancer survivors' self‐efficacy, which includes confidence and sense of control over their health, or simple tasks such as calling their doctors if have any concerns related to their cancer or treatment. In contrast to the above findings, another study recommended that improvement of self‐efficacy could be achieved by using written care plans supported by verbal explanation, which led to a lower frequency of emergency room visits.^51^

The impact of wellbeing on survival requires further exploration to indicate endpoints for intervention, in addition to achieving longer survival. A review of 104 citations looked at prognostic indicators of survival in cancer patients and found positive relationships between quality of life and survival duration.^52^ A meta‐analysis of 17 studies suggested that enhancement of emotional wellbeing predicted long‐term prognosis of physical illness.^53^ The study found patients with higher baseline levels of emotional wellbeing have better recovery and survival rates than those without. These findings suggest a patient‐centred intervention for AI/ACC needs to include content and format that supports patient wellbeing as this may have an impact on their survival.

### 4.3 Strengths

This is the first review to identify the burden of disease and treatment on this population, and the paucity of patient experience evidence in the management of AI/ACC. The search terms for AI and ACC had many variations which may have impacted on the search outcomes.

### 4.4 Limitation

Low‐quality evidence such as the case reports were included as they contributed to a better understanding on the lived experience of patients under study. Involvement of multiple reviewers at title/abstract screening stages could enhance the rigour of studies selection. It was challenging to assess the strength of evidence related to research questions of this study as they were limited throughout AI/ACC patients' journey, and quantitative studies failed to address the complex relationship between AI and ACC despite the evidence in case studies. The lack of studies from countries which do not have dominance in white Caucasian populations was noted. Studies from those populations could have contributed to wider understanding about the impact of ACC.

### 4.5 Recommendations for research and clinical practice

Cancer survivorship is considered to have three phases of survival including dealing with one's own sense of mortality, engaging with a period of surveillance, aiming to lower the risk of recurrence and other risks related to oncology late effects.^35^ Healthcare professionals should recognize survivorship phases including side effects and symptoms monitoring to optimize care, just as they might use mitotane levels to optimize patient's progression free survival. In the context of ACC, the development of AI and the shared features of AI such as nausea and tiredness, with the disease and treatment related symptoms, add an additional dimension to the complexity of management. The relative paucity of data indicates a need to evaluate a self‐management programme for AI, although acknowledging that the timing of such an intervention would need to align with the phase of survival.

A recognition that adrenal crisis related to AI management in ACC might be a preventable condition is an important understanding for healthcare professionals. The impact of AI/ACC on patient's wellbeing from diseases, medication and therapeutic regimes places a considerable burden on their survivorship. Clinicians should be alerted to these burdens and the patients' capacity to withstand the burden and concord with treatment recommendations requires careful assessment and supportive care provision. Finally, the relationship between wellbeing and survivorship of ACC warrants further investigation to understand the poor survival of this cohort.

## 5 CONCLUSION

Patients with AI/ACC face many challenges. Their lived experience remains poorly understood and they are faced with burdens that may impact on their wellbeing from diagnosis and throughout their treatment pathways. Carers of patients' living with ACC play a crucial role in supporting their wellbeing and lived experience; improved structure is required to support carers' efforts. Qualitative interviews are needed to illuminate the lived experience, wellbeing and needs of patients living with this condition. Combining AI and ACC is particularly difficult for patients in terms of the challenges it presents, and the impact of those challenges on their wellbeing. A better understanding of those challenges would enable healthcare professionals to provide much more person‐centred and ameliorative care.

## AUTHOR CONTRIBUTIONS

**Phillip Yeoh:** Conceptualization (equal); data curation (equal); formal analysis (equal); funding acquisition (equal); investigation (equal); methodology (equal); project administration (equal); resources (equal); validation (equal); visualization (equal); writing – original draft (equal); writing – review and editing (equal). **Wladyslawa Czuber‐Dochan:** Conceptualization (equal); data curation (equal); formal analysis (equal); investigation (equal); methodology (equal); supervision (equal); validation (equal); visualization (equal); writing – review and editing (equal). **Simon Aylwin:** Supervision (equal); writing – review and editing (equal). **Jackie Sturt:** Conceptualization (equal); data curation (equal); formal analysis (equal); investigation (equal); methodology (equal); supervision (equal); validation (equal); visualization (equal); writing – review and editing (equal).

## CONFLICT OF INTEREST

The authors have no financial or non‐financial competing interests to declare.

## Supporting information


Appendix S1

## Data Availability

Data sharing is not applicable to this article as no new data were created or analyzed in this study.
