# Supplementary material for: Lived experience of people with adrenocortical carcinoma and associated adrenal insufficiency
Source: Endocrinol Diabetes Metab. 2022 Jun 6;5(4):e341. doi: 10.1002/edm2.341 (PMC9258998; doi:10.1002/edm2.341)
Supplement: Supplementary file 1 — Appendix S1 [file EDM2-5-e341-s001.docx]

**Supplementary table 1: Search strategy table**

Medical Subject Heading (MeSH) that included all sub-heading, sub-terms, truncation, Boolean operator, free texts

| **Population/Patient**  **/Problem** | **Intervention** | **Comparison** | **Outcomes** |
| --- | --- | --- | --- |
| **Key words** |  |  |  |
| **ACC** | **Management** | **Adrenal insufficiency** | **Living experience** |
| **Adrenocortical carcinoma** | **Treatment** | **Adrenal insufficient** | **Lived experience** |
| **Adrenal cortical carcinoma** | **Care pathway** | **Adrenal insufficienc*** | **Day to day experience** |
| **Adrenocortical neoplasm** | **Health management** | **hypopituitarism** | **Needs** |
| **Adrenal cortex cancer** | **Clinical management** | **hypopit** | **Survival** |
| **Adrenal cortex carcinoma** | **Clinical care** | **hypocortisolism** | **Quality of life** |
| **Adrenal cortex neoplasm** | **Clinical intervention** | **Addison’s** | **Self-management** |
| **adrenocortical adj5 carcinoma*** | **Mitotane** | **Addison*** |  |
| **adrenocortical adj5 tumo?r*** | **Cisplatin** | **Congenital adrenal hyperplasia** |  |
|  | **Etoposide** | **CAH** |  |
|  | **Doxorubicin** | **Adrenal crisis** |  |
|  | **Immunotherapy** | **Addisonian crisis** |  |
|  | **Immunotherap*** | **Adrenal failure** |  |
|  | **Adjuvant therapy** | **hypocortisolaemia** |  |
|  | **Adjuvant therap*** | **Low cortisol** |  |
|  | **Surgery** | **ACTH deficiency** |  |
|  | **Adrenalectomy** | **Cushing’s** |  |
|  | **Adrenalectom*** | **Cushing*** |  |

**Supplementary table 2: Appraisal for 15 studies using Mixed Method Appraisal Tool (MMAT) version 2018^20^**

MMAT consisted of appraising five categories of study design with each of these categories having five screening questions with the reviewer required to score ‘Yes’, ‘No’ or ‘Can’t tell’ against each question. Each criterion met is scored with ‘Yes’ and the number of ‘Yes’ responses determined the quality of the included studies

| **Study designs** | **Methodological quality criteria** | **Yes** | **No** | **Can’t tell** |
| --- | --- | --- | --- | --- |
| **Quantitative non-randomized (non- randomized controlled trials, cohort study, case-control study, cross-sectional analytic study)** | Q1: Are the participants representative of the target population? |  |  |  |
|  | Q2: Are measurements appropriate regarding both the outcome and exposure/intervention? |  |  |  |
|  | Q3: Are there complete outcome data? |  |  |  |
|  | Q4: Are the confounders accounted for in the design and analysis? |  |  |  |
|  | Q5: During the study period, is the intervention/exposure administered as intended? |  |  |  |
| **Quantitative descriptive (survey, case series, case report)** | Q1: Is the sampling strategy relevant to address the research question? |  |  |  |
|  | Q2: Is the sample representative of the target population? |  |  |  |
|  | Q3: Are the measurements appropriate? |  |  |  |
|  | Q4: Is the risk of nonresponse bias low? |  |  |  |
|  | Q5: Is the statistical analysis appropriate to answer the research questions? |  |  |  |

| **Studies** | **QUANTITATIVE NON RANDOMIZED STUDIES** | | | | | **QUANTITATIVE DESCRIPTIVE STUDIES** | | | | | **TOTAL SCORE** | |
| --- | --- | --- | --- | --- | --- | --- | --- | --- | --- | --- | --- | --- |
|  | **Q1** | **Q2** | **Q3** | **Q4** | **Q5** | **Q1** | **Q2** | **Q3** | **Q4** | **Q5** |  |  |
| **Haak et al 1994** | YES | YES | YES | YES | YES |  |  |  |  |  | 100% |  |
| **Willianson et al 1999** | YES | YES | YES | YES | YES |  |  |  |  |  | 100% |  |
| **Abraham et al 2002** | YES | YES | YES | YES | YES |  |  |  |  |  | 100% |  |
| **Berruti et al 2005** | YES | YES | YES | YES | YES |  |  |  |  |  | 100% |  |
| **Zancanella et al 2006** | YES | YES | YES | YES | NO |  |  |  |  |  | 80% |  |
| **Daffara et al 2008** | YES | YES | YES | YES | YES |  |  |  |  |  | 100% |  |
| **Sperone et al 2010** | YES | YES | YES | YES | YES |  |  |  |  |  | 100% |  |
| **Lacroix 2010** |  |  |  |  |  | YES | YES | YES | YES | NO | 80% |  |
| **Meuclère-Denost et al 2012** | YES | YES | YES | YES | YES |  |  |  |  |  | 100% |  |
| **Terzolo et al 2013** | YES | YES | YES | YES | YES |  |  |  |  |  | 100% |  |
| **Lerario et al 2014** | YES | YES | YES | YES | NO |  |  |  |  |  | 80% |  |
| **Fancellu et al 2014** |  |  |  |  |  | YES | YES | YES | YES | NO | 80% |  |
| **Kanjanapan et al 2015** |  |  |  |  |  | YES | YES | YES | YES | NO | 80% |  |
| **Head et al 2019** |  |  |  |  |  | YES | YES | YES | YES | YES | 100% |  |
| **Muratori et al 2020** |  |  |  |  |  | YES | YES | YES | YES | NO | 80% |  |

**Supplementary table 3: Characteristics of the included studies (in chronological order)**

| Author, year,  Country, study design | Aim of study | Sample characteristic | Outcomes assessed and measures used | Main results/theme |
| --- | --- | --- | --- | --- |
| Haak et al  1994  The Netherlands  Cross sectional study | To evaluate the relevance of mitotane serum levels greater than 14mg/L and other influences on survival | **Population:** Patients taking mitotane  **Sample size:** 96  **Gender (M/F):** 40/56  **Age (median, range):** 44.4 (1-78)  **Presentations**  Pain 47% | Mitotane therapy and mitotane serum levels low is <14mg/l and high is >14mg/l | 59% experienced hormonal excess, 47% presented with pain and 29% had palpable abdominal mass, 47/84 (56%) were operable leading to changes in physical appearance and health, affected physical, mental, and psychological well-being. In those taking mitotane, 30/62 (48%) was able to achieve high mitotane level. 10/96 (10%) discontinued mitotane and up to 90% of them reported toxicity effects such as anorexia, nausea, vomiting, diarrhoea, thrombocytopenia leading to bleeding and bruising, CNS and neuropsychiatric symptoms. Gastrointestinal symptoms were commonly reported and presented at early phase of mitotane treatment. All patients with inoperable disease died within 18 months. |
| Williamson et al 2000  USA  Cohort study | To evaluate the response rate and toxic effects to mitotane after disease progression on etoposide and cisplatin | **Population:** Patients with advanced/metastatic ACC  **Sample size:** 45  **Gender (M/F):** 23/22  **Age in 2 groups:**  Group 1 median age: 46.5 (range 12-72)  Group 2 median age: 30 (range 18-46)  **Race:**  White N = 39 (87%)  Black N = 4 (8.8%)  Hispanic N = 2 (4.4%)  **Self-care status**  Grade 0-1 N = 40  Grade 2 N = 5 | **Southwest Oncology Group self-care performance status**  Grade 1 = restricted  Grade 2 = unable to work  **Toxicity was graded using National Cancer Institute (NCI) Common**  **Toxicity Criteria**  Grade 1 Mild  Grade 4 Disabling | 5/45 (11%) were unable to work and it was not clear the number of 40/45 (89%) cohorts had physical limitation.  2/45 ((4%) died of infection and respiratory distress. Consequent to mitotane toxicity, up to 82% of patients experienced mild to disabling category of nausea, vomiting, anaemia, thrombocytopenia, fatigue, feeling weak and had paraesthesia with profound impact on physical health, quality of life, well-being, and self-care. |
| Abraham et al  2002  USA  Cohort study | To determine the efficacy of doxorubicin, vincristine and etoposide with oral mitotane therapy | **Population:** Patients with metastatic ACC  **Sample size:** 36  **Gender (M/F):** 11/25  **Age (median, range):** 44.4, 23-70  **Self-care performance status**  Grade 1 N = 36 | **ECOG self-care performance status**  Grade 1 = restricted  Chemotherapy was administered in every 3-week cycles  **Toxicity was graded using National Cancer Institute (NCI) Common**  **Toxicity Criteria**  Grade 1 Mild  Grade 2 Moderate | All cohorts were restricted in their physical activities. They had hormone excess, various metastasis sites and numerous adjuvant therapy affecting physical appearance, mental, and psychological well-being.  Patients required a median of 5.6 cycles of chemotherapy to achieve a median survival of 34.3 months. Median survival for patients whose tumour did not respond to chemotherapy was 11.6 months.  Up to 66% experienced chemotherapy toxicity such as neutropenia which put patients at risk to infection. Other side effects were anaemia, fever, nausea/vomiting, and thrombocytopenia. Consequent to mitotane toxicity, up to 47% of patients experienced gastrointestinal, neurological, physical vitality symptoms at mild to moderate levels presented continuously without relief affecting the quality of life and wellbeing. |
| Berruti et al  2005  Italy  Cohort study | To investigate the activity of etoposide, doxorubicin and cisplatin plus mitotane | **Population:** Patients with advanced ACC  **Sample size:** 72  **Gender (M/F):** 24/48  **Age (median, range):** 50, 18-73  **Self-care performance status**  Grade 0 N = 31  Grade 1 N = 28  Grade 2 N = 11  Grade 3 N = 2 | **ECOG self-care performance status**  Grade 1 = restricted  Grade 2 = unable to work  Grade 3 = Limited self-care  **Toxicity was graded using WHO criteria:**  Grade 1 Mild  Grade 3 Severe | 39% of cohorts were restricted in physical activity, 15% unable work and 3% were limited in self-care. 48/70(68%) had hormone hypersecretion with 29/72(41%) affected physical appearance, mental and psychological wellbeing.  52.8% completed treatment with a median of 6 chemotherapy cycles to achieve 48.6% objective regression and overall survival of up to 47.7 months.  Consequent to chemotherapy + mitotane treatment, up to 65/72 (90%) experienced gastrointestinal, haematological, hepatic, neurological, renal, and cardiac toxicity from mild to severe grade resulted in 6.9% stopping mitotane. |
| Zancanella et al 2006  Brazil  Cohort study | To define a mitotane dose that maintains therapeutic plasma levels (TL) between 14 and 20 mg/mL | **Population:** Children with ACC  **Sample size:** 11  **Gender (M/F):** 3/8  **Age (range):** 2-11.2 years | Mitotane toxicity symptoms was reported but not graded | All patients experienced mitotane toxicity with gastrointestinal symptoms. Others were neurologic alterations, physical appearance changes, and hypertensive encephalopathy.  One patient died from adrenal crisis due to carer did not increase the glucocorticoid dose during sickness. 5/11 (45%) experienced adrenal crisis during the study. Carers needed to provide direct care and support to patients for them to adhere to ACC treatments. |
| Daffara et al  2008  Italy  Cohort study | To assess the unwanted effects of adjuvant mitotane treatment | **Population:** Patients with ACC  **Sample size:** 17  **Gender (M/F):** 7/10  **Age (median, range):** 36, 22-58 | **Toxicity was graded using National Cancer Institute (NCI) Common**  **Toxicity Criteria**  Grade 1 Mild  Grade 2 Moderate  Grade 3 Severe | 37% experienced tumour recurrence, 3/17 (17%) died of ACC progression and it took 9 months for them to achieve therapeutic mitotane level. Consequent to mitotane toxicity, 11.8 - 71% experienced gynecomastia, fatigue, nausea/vomiting, male impotence, orthostatic hypotension, anorexia, diarrhoea, leukopenia, confusion or vertigo/dizziness and ataxia. All patients developed AI, 31% had hypothyroidism, 47% had hypercholesterolaemia, 57% of men had low testosterone and required treatments. These effects had impacted the physical well-being and quality of life however, it was not clear how they coped and managed their comorbidities. |
| Sperone et al 2010  Italy  Cohort study | To assess the activity and toxicity of chemotherapy regimen consist of gemcitabine or capecitabine plus metronomic fluoropyrimidines | **Population:** Patients with advanced ACC  **Sample size:** 28  **Gender (M/F):** 12/16  **Age (median, range):** 45, 23-72  **Self-care performance status**  Grade 0 N = 15  Grade 1 N = 10  Grade 2 N = 3 | **ECOG self-care performance status**  Grade 1 = restricted  Grade 2 = unable to work.  **Toxicity was graded using WHO criteria:**  Grade 1 Mild  Grade 2 Moderate  Grade 3 Severe | 36% of cohorts were restricted in physical activity with 11% unable to work. Up to 85% had adjuvant therapy, 32% had additional second-line regimens, 50% had hormonal excess leading to changes in their health, appearance, and impact in self-care and quality of life. Cohorts received a median treatment of 6 months and toxicity of severe or disabling effects were presented in leukopenia, mucositis, or thrombocytopenia. While 46.4% reported clinical benefit after 4 months of treatment, 54% of cohorts experienced fatigue, gastrointestinal or paraesthesia, anaemia, thrombocytopenia, conjunctivitis, neurologic symptoms, or fever with up to 14.3% required dose reduction, delay or temporary discontinuation due to toxicity effect, impairment of quality of life, and self-care.  Overall survival of participants were 9.8 months. |
| Lacroix  2010  Canada  Case report | To describe the challenges of ACC clinical management | **Population:** A patient with ACC  **Gender:** F  **Age:** 22 |  | Presented with virilisation, amenorrhea and hormone excess led to changes in physical appearance.  Required multiple surgeries for recurrences and mitotane adjuvant therapy followed by chemotherapy. AI and mitotane toxicity management were challenging required constant glucocorticoid and mitotane titration. She was given MDT and palliative care support when her prognosis deteriorated but it was not clear what the intervention supports were provided. |
| Meuclère-Denost et al  2012  France  Cohort study | To evaluate the performance of highest tolerated mitotane dose administered within 2 weeks and maintenance therapy over 4 weeks | **Population:** All patients who were started on high dose mitotane therapy for treatment of ACC in a single centre  **Sample size:** 22  **Gender (M/F):** 8/14  **Age (median, range):** 59, 25-73  **Self-care performance status**  Grade 1 N = 8  Not stated N=14 | **WHO self-care performance status**  Grade 1 = restricted  **Toxicity was graded using National Cancer Institute (NCI) Common**  **Toxicity Criteria**  Grade 1 Mild  Grade 4 Disabling | 36% had restricted physical activity, 68% had hormone excess, all patients had surgery including 54% had nephrectomy, 41% received mitotane as palliative treatment and only 45% were able to achieve therapeutic mitotane level. These experiences and outcomes had impacts on physical appearance, quality of life, self-care, physical, mental, and psychological well-being of patients. In 11/22 (50%) who transiently discontinued mitotane due to toxicity, 18-91% experienced mild to disabling grade in gastrointestinal, hepatic, haematological, dermatological, neurological anorexia, gynaecomastia, and fatigue symptoms. 4/22(18%) permanently discontinued due to tumour progression. These challenges had impacts on patients’ quality of life. All patient received normal salt diet and AI education, but it was not clear what was the intervention involved. |
| Terzolo et al  2013  Italy/Germany/The Netherlands  Cohort study | Retrospective study to compare recurrence free survival (RFS) patients who reached and maintained mitotane  concentrations ≥14 mg/l vs patients who did not | **Population:** Patients with ACC who had radical resection and monitored adjuvant mitotane treatment  **Sample size:** 122  **Gender (M/F):** 53/69  **Age (median, range):** 45, 16-76 | **Toxicity was graded using National Cancer Institute (NCI) Common**  **Toxicity Criteria**  Grade 3 Severe  Grade 4 Disabling | 70% had mitotane measurements every 3 months and median duration of mitotane treatment was 25 months. Consequent to effects of mitotane, only 53% were able to achieve target mitotane concentration despite that its significantly prolonged recurrent free survival. 24.5% discontinued mitotane treatment by choice and of those who temporary discontinued, 9% experienced severe or disabling toxicity. Mild and moderate toxicity were reported in 21.3% with neurologic symptoms, 25.3% with hepatic symptoms and 36% with gastrointestinal symptoms. 47.5% experienced ACC recurrence. 27% die from ACC. These outcomes would have an impact on physical, mental, and psychological wellbeing. |
| Lerario et al  2014  USA  Cohort study | To assess the efficacy of the combination of the IGF1R inhibitor cixutumumab (IMC-A12) in association with mitotane | **Population:** Adults with metastatic ACC  **Sample size:** 20  **Gender (M/F):** 13/7  **Age (median, range):** 50.2, 21.9-79.6  **Self-care performance status**  Grade: 0 N = 12  Grade 1 N = 8 | **ECOG self-care performance status**  Grade 1 = restricted | 8/20 (40%) were unable to perform physical strenuous activity. Toxicity effects were neurological, psychiatric, and gastrointestinal. There was one (5%) death with multiorgan failure, and 2 (10%) severe cases of hyperglycaemia and study was terminated. |
| Fancellu et al 2014  Italy  Case report | A case study to describe the experience of a person with ACC | **Population:** Patient with ACC  **Gender:** M  **Age:** 41 |  | Presented 6 months history of gynaecomastia, weight loss, physical pain, gastrointestinal symptoms, constipation and previously refused genetic testing despite sister had MEN1 which predispose him to develop ACC. He had Cushing’s disease, high estradiol, low testosterone levels and this would have affected his physical appearance, quality of life, mental and psychological well-being. He had bilateral adrenalectomy for a 3.3 kg tumour followed by glucocorticoid replacement therapy and mitotane adjuvant therapy without any important side effects with improvement in gynecomastia afterwards. |
| Kanjanapan et al 2015  Australia  Case report | To illustrate the clinical management of patients diagnosed with ACC with multidisciplinary team approach | **Population:** A patient with ACC  **Gender:** F  **Age:** 47 |  | Presented with abdominal pain, virilisation of hairlines, androgen excess which affected her physical appearance and physical well-being. She had adrenalectomy followed by mitotane and glucocorticoid replacement therapy. At early stage, she had an adrenal crisis precipitated by missed glucocorticoid dose. Steroid education on sick day management was reinforced. Following two years of mitotane therapy, she elected to stop mitotane due to its toxicity and remained on glucocorticoid replacement. 7 months later, she had another adrenal crisis precipitated by viral illness resulted in cardiac arrest and death. It was not clear what did her steroid education intervention involved. |
| Head et al 2019  USA  Case series | Efficacy of immunotherapy (pembrolizumab) in combination with mitotane | **Population:** Patient with metastatic ACC  **Sample size:** 6  **Gender:** F  **Age (mean, range): 44,** 24-65  **Performance status**  Grade 0 N = 1  Grade 1 N = 5 | **ECOG self-care performance status**  Grade 1 = restricted  Treatment toxicity was graded but it was not cleared which tool was used | 83% were unable to perform physical strenuous activity. 50% had hormonal excess and 2/3 of them had more than one surgery. Consequent to treatment, up to 66.7% patients had grade 2 or above toxicity in fatigue or nausea, rash or diarrhoea, decreased appetite, headache, neuropathy, pruritis, dyspnoea, rectal bleeding or dehydration, pneumonitis, hepatitis, or mouth sores. All patients had hypothyroidism. The presenting symptoms, care pathways and toxicity effects would have an impact on the physical appearance, physical and mental well-being of patients. It was not clear how they coped with chronic hypothyroidism. |
| Muratori et al 2020  Italy  Case study | To describe symptomatic AI diagnosed three years after discontinuation of mitotane | **Population:** Patient with ACC  **Gender:** M  **Age:** 37 |  | Presented with intermittent fever, abdominal pain, hypertension, diabetes mellitus, obesity, physical changes with left retroperitoneal mass of 18 x 12 cm. Had surgery, radiotherapy, adjuvant mitotane and glucocorticoid replacement therapy. Mitotane blood levels were inconsistently within therapeutic range due to poor compliance and two years later patient choose to stop mitotane. Glucocorticoid was titrated and stopped. Few months later, had peanut anaphylaxis event and was treated with glucocorticoid, asthenia ensued and became severe. Blood tests showed low cortisol and recommenced on glucocorticoid replacement therapy. Subsequent test confirmed partial AI which was 3 years after stopping mitotane. However, glucocorticoid dose was gradually titrated downward and second ACTH stimulated test confirmed full adrenal recovery and subsident of AI. |
